# Changes in programmed death-ligand 1 expression during cisplatin treatment in patients with head and neck squamous cell carcinoma

**DOI:** 10.18632/oncotarget.18542

**Published:** 2017-06-16

**Authors:** Chan-Young Ock, Sehui Kim, Bhumsuk Keam, Soyeon Kim, Yong-Oon Ahn, Eun-Jae Chung, Jin-Ho Kim, Tae Min Kim, Seong Keun Kwon, Yoon Kyung Jeon, Kyeong Chun Jung, Dong-Wan Kim, Hong-Gyun Wu, Myung-Whun Sung, Dae Seog Heo

**Affiliations:** ^1^ Department of Internal Medicine, Seoul National University Hospital, Seoul, Korea; ^2^ Department of Pathology, Seoul National University Hospital, Seoul, Korea; ^3^ Cancer Research Institute, Seoul National University College of Medicine, Seoul, Korea; ^4^ Department of Otorhinolaryngology, Seoul National University Hospital, Seoul, Korea; ^5^ Department of Radiation Oncology, Seoul National University Hospital, Seoul, Korea

**Keywords:** programmed death-ligand 1, head and neck squamous cell carcinoma, cisplatin

## Abstract

Programmed death-ligand 1 (PD-L1) expression is regarded as a predictive marker for anti-PD-1/PD-L1 therapy. The purpose of study was to explore the changes in PD-L1 expression in head and neck squamous cell carcinoma (HNSCC) during treatment. Paired HNSCC tissues prior to and after cisplatin-based treatment were evaluated to determine PD-L1 protein expression by immunohistochemistry. Among the 35 HNSCC patient samples, PD-L1 expression status changed after treatment in 37.1% (13/35) of samples. Among the 13 patients whose baseline PD-L1 was negative, PD-L1 expression was increased in 9 cases (69.2%) and remained negative in 4 cases (30.8%, *P* = 0.003). Patients exposed to cisplatin generally showed PD-L1 up-regulation (83.3%, *P* = 0.037) compared to those not exposed to cisplatin (57.1%, *P* = 0.072). To validate these findings *in vitro*, changes in PD-L1 expression in HNSCC cell lines (Detroit-562, PCI-13, SNU-1041, SNU-1066, SNU-1076, and FaDu) were analyzed by western blotting and flow cytometry after treatment with cisplatin and interferon-gamma. In HNSCC cell lines, PD-L1 expression was significantly up-regulated after cisplatin, along with phosphor-MAPK/ERK kinase up-regulation. In conclusion, PD-L1 expression in HNSCC may be altered during cisplatin treatment, activating the MAPK/ERK kinase pathway.

## INTRODUCTION

Programmed death-ligand 1 (PD-L1) expression in tumors was recently introduced as a novel immunologic therapy for cancer [1, 2]. Cancer develops various strategies for evading host anti-cancer immunologic attack, such as escaping immune surveillance by up-regulating PD-L1, which can induce T cell anergy and immune escape by interacting programmed death 1 (PD-1) and PD-L1. Recently, immune checkpoint-blocking agents, which target PD-1/PD-L1 pathways in T cells to enhance anti-tumor immune responses, have shown promising anti-tumor activity and provided a new paradigm in cancer therapy [1, 2]. However, more than half of patients were insensitive to this agent in a previous study. Therefore, defining a predictive biomarker for PD-1/PD-L1 blocking agents is very important. Various biomarkers such as mutational burden [3, 4], the presence of tumor-infiltrating lymphocytes [5, 6], and interferon-gamma signature [7] have been suggested for predicting the response to anti-PD-1/PD-L1 inhibitors; among these, PD-L1 expression in tumor cells is a promising and simple predictive biomarker [8, 9].

The clinical use of PD-L1 as a predictive marker for PD-L1/PD-1 blocking agents is controversial for several reasons. The most important reason is that the expression of PD-L1 differs during the clinical course and tumor evolution process. Increasing *in vitro* evidence has shown that the expression of PD-L1 in tumors changes in response to various exogenous signals including interferon-gamma, radiotherapy, or chemotherapeutic agents [10–12]. PD-L1 was up-regulated after the development of acquired resistance to gefitinib in epidermal growth factor receptor-mutant non-small cell lung cancer (NSCLC), involving the epithelial-mesenchymal transition (EMT) and MAPK/ERK kinase (MEK) pathway [13, 14]. Interestingly, we previously reported that PD-L1 up-regulation was also associated with EMT in HNSCC [15]. Therefore, PD-L1 changes during the treatment course may be also evident in HNSCC, but few studies have examined this.

We hypothesized that PD-L1 expression changes during treatment in HNSCC patients. Thus, the aim of this study was to determine changes in PD-L1 expression during cisplatin chemotherapy in HNSCC cancer patients.

## RESULTS

### PD-L1 expression changes in HNSCC patient samples

Of the 35 HNSCC patients included in this study, baseline tumor tissue of 22 patients (62.9%) had PD-L1-positive tumors, while the other 13 patients (37.1%) had PD-L1-negative tumors (Table 1). Clinical features including tumor location of the oropharynx and p16-status did not significantly differ according to baseline PD-L1 status, although baseline PD-L1-positive tumors showed a relatively high proportion of oropharyngeal tumors (7 of 22, 31.8% in PD-L1-positive vs. 1 of 13, 7.7% in PD-L1-negative) and p16-positive tumors (6 of 22, 27.3% in PD-L1-positive vs. 1 of 13, 7.7% in PD-L1-negative).

**Table 1:** Patient characteristics

PD-L1	Before treatment	Negative	Negative	Positive	Positive	All	*P*	*P*
	After treatment	Negative	Positive	Negative	Positive			
		*N* = 4	*N* = 9	*N* = 4	*N* = 18	*N* = 35	Baseline PD-L1^*^	4 groups
Age	Median years (range)	68 (52–78)	63 (51–70)	42 (26–76)	62 (16–75)	63 (16–78)	0.260	0.443
Sex	Men, N (%)	3 (75.0)	8 (88.9)	4 (100.0)	16 (88.9)	31 (88.6)		
	Women, N (%)	1 (25.0)	1 (11.1)	0 (0)	2 (11.1)	4 (11.4)	0.478	0.860
Smoking	Non-smoker, N (%)	2 (50.0)	5 (55.6)	3 (75.0)	14 (77.8)	24 (68.6)		
	Ex/Current-smoker, N (%)	2 (50.0)	4 (44.4)	1 (25.0)	4 (22.2)	11 (31.4)	0.144	0.528
ECOG	0, N (%)	0 (0)	1 (11.1)	1 (25.0)	8 (44.4)	10 (28.6)		
	1, N (%)	4 (100)	8 (88.9)	3 (75.0)	10 (55.6)	25 (71.4)	0.055	0.078
Location	Oropharynx, N (%)	0 (0)	1 (11.1)	2 (50.0)	5 (27.8)	8 (22.9)		
	Non-oropharynx^*^, N (%)	4 (100)	8 (88.9)	2 (50.0)	13 (72.2)	27 (77.1)	0.108	0.346
p16	Positive, N (%)	0 (0)	1 (11.1)	3 (75.0)	3 (16.7)	7 (20.0)		
	Negative, N (%)	4 (100)	8 (88.9)	1 (25.0)	15 (83.3)	28 (80.0)	0.170	0.058
Pathology	SqCC P/D, N (%)	2 (50.0)	3 (33.3)	1 (25.0)	6 (33.3)	12 (34.3)		
	SqCC M/D, N (%)	1 (25.0)	5 (55.6)	2 (50.0)	2 (11.1)	10 (28.6)		
	SqCC W/D, N (%)	1 (25.0)	1 (11.1)	1 (25.0)	8 (44.4)	11 (31.4)		
	Non-keratinizing type, N (%)	0 (0)	0 (0)	0 (0)	2 (11.1)	2 (5.7)	0.189	0.375
Stage	I, N (%)	1 (15.0)	0 (0)	1 (25.0)	3 (16.7)	5 (14.3)		
	II, N (%)	0 (0)	0 (0)	0 (0)	1 (5.6)	1 (2.9)		
	III, N (%)	3 (75.0)	2 (22.2)	0 (0)	2 (11.1)	7 (20.0)		
	IVA, N (%)	0 (0)	7 (77.8)	3 (75.0)	12 (66.7)	22 (62.9)	0.187	0.254
Definitive treatment	Concurrent chemoradiotherapy, N (%)	3 (75.0)	4 (44.4)	1 (25.0)	6 (33.3)	14 (40.0)		
	Surgery, N (%)	1 (25.0)	5 (55.6)	3 (75.0)	12 (66.7)	21 (60.0)	0.177	0.507
Cisplatin treatment	No (never), N (%)	3 (75.0)	4 (44.4)	3 (75.0)	8 (44.4)	18 (51.4)		
	Yes (ever), N (%)	1 (25.0)	5 (55.6)	1 (25.0)	10 (55.6)	17 (48.6)	0.552	0.212
Interval between harvesting tissues	Median months (range)	12.4 (1.0–44.5)	14.9 (1.5–33.4)	9.4 (3.3–11.6)	8.7 (1.8–39.9)	11.6 (1.0–44.5)	0.246	0.603
Overall survival	Median months (95% CI)	25.0 (15.9–NR)	50.1 (22.6–NR)	NR (35.7–NR)	43.7 (30–NR)	50.1 (32.6–NR)	0.451	0.859
	3-year survival rate	37.5%	72.9%	66.7%	61.5%	63.0%		
	5-year survival rate	37.5%	18.2%	0%	49.2%	38.6%		
Median follow-up	Median months (range)	75.1 (46.3–88.2)	62.4 (27.4–119.6)	73.7 (23–111.7)	45.1 (29–104.5)	62.4 (23–119.6)	0.306	0.641

^*^P value of comparison of PD-L1-negative before treatment (1st and 2nd columns) and PD-L1-positive before treatment (3rd and 4th columns).

^**^ Non-oropharynx included hypopharynx, larynx, nasal cavity, paranasal sinus, and oral cavity, which were not significant according to PD-L1 positivity.

Abbreviation: ECOG, Eastern Cooperative Oncology Group performance status; SqCC, Squamous cell carcinoma; P/D, poorly-differentiated squamous cell carcinoma; M/D; moderate-differentiated squamous cell carcinoma; W/D, well-differentiated squamous cell carcinoma; CI, confidence interval; NR, not reached.

**Figure 1:**
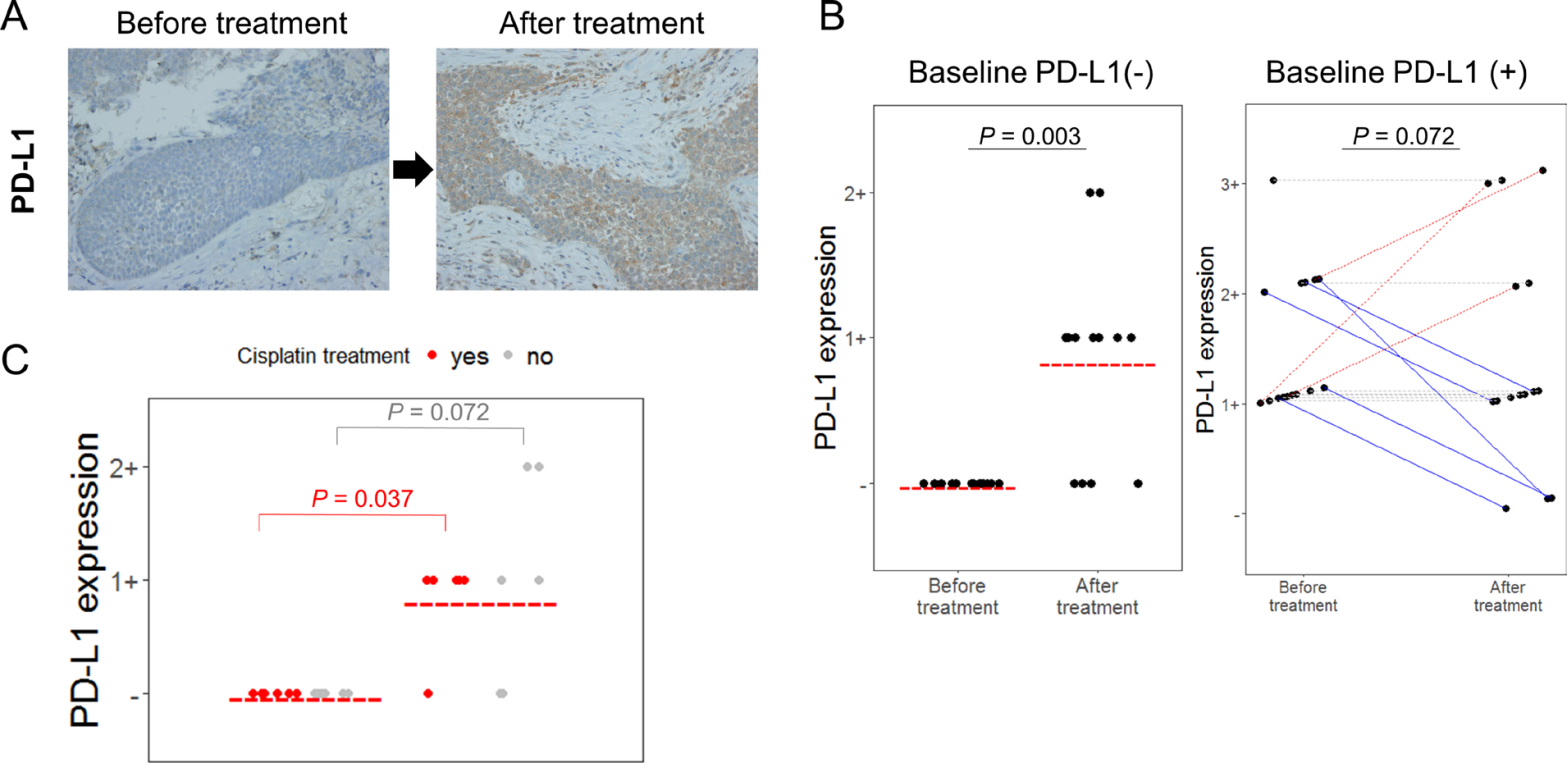
PD-L1 changed after treatment in HNSCC patient samples (**A**) Representative PD-L1 immunochemical staining (×400). PD-L1 expression was increased after treatment. (**B**) Changes in PD-L1 expressions before treatment (baseline) and after treatment in 35 HNSCC patients are shown. Each dot represents PD-L1 expression positivity, and red bar represented indicates PD-L1 expression positivity in each group. For the right side graph, each line connects the same individual. Red dash lines represent PD-L1 positivity is increased, while blue solid lines represents PD-L1 positivity is decreased. (**C**) Among baseline PD-L1-negative patients, PD-L1 positivity measured by immunohistochemistry was more significantly up-regulated in cisplatin-treated patients (red dot) compared to those who were not exposed to cisplatin (grey dot). *P* values were noted for comparison of before treatment (baseline) PD-L1 positivity and after treatment PD-L1 positivity in each group of cisplatin-treated patients (red) and cisplatin-naïve patients (grey).

During the treatment course, PD-L1 changes in various ways. Of the 13 patients who were PD-L1-negative prior to treatment, 9 cases (69.2%) showed up-regulated PD-L1 expression after treatment (*P* = 0.003). In contrast, 4 of 22 patients (18.2%) who were initially PD-L1-positive tumor showed decreased PD-L1 expression (*P* = 0.072, Figure 1A–1B). Interestingly, most patients who underwent cisplatin treatment as induction chemotherapy or concurrent chemoradiotherapy (CCRT) showed up-regulated PD-L1 expression in their post-treatment tumor tissue (*P* = 0.037, Figure 1C and Table 2). As the previous study showed that radiotherapy was associated with PD-L1 up-regulation [16], we also analyzed if radiotherapy was also associated with PD-L1 changes. In baseline PD-L1 negative patients, radiotherapy was significantly associated with PD-L1 up-regulations (*P* < 0.001 versus *P* = 0.072, Supplementary Figure 1).

**Table 2:** Summary of PD-L1-negative HNSCC patient treated with cisplatin

HNSCC type	Sex/Age	p16 status	Brief history	Baseline tissue	Baseline PD-L1	Post-treatment tissue	Post PD-L1
hypopharynx	F/66	(−)	Induction chemotherapy → definitive CCRT → salvage operation	Before induction chemotherapy, primary tumor	(−)	On salvage operation, recurred tumor	(−)
Hypopharynx	M/63	(−)	Induction chemotherapy → definitive CCRT → salvage operation	Before induction chemotherapy. primary tumor	(−)	On salvage operation, recurred tumor	(1+)
Pyriform sinus	M/62	(−)	Induction chemotherapy → definitive operation → post-RT → salvage operation	Before induction chemotherapy. primary tumor	(−)	On salvage operation, recurred tumor	(1+)
Nasal cavity	M/60	(−)	Definitive CCRT → salvage operation	Before CCRT, primary tumor	(−)	On salvage operation, recurred tumor	(1+)
Larynx	M/66	(−)	Definitive operation → post-CCRT → salvage operation	On definitive operation, primary tumor	(−)	On salvage operation, recurred tumor	(1+)
Hypopharynx	M/69	(−)	Induction chemotherapy → definitive CCRT → salvage operation	Before induction chemotherapy. primary tumor	(−)	On salvage operation, recurred tumor	(1+)

Abbreviation: HNSCC, head and neck squamous cell carcinoma; CCRT, concurrent chemoradiotherapy; RT, radiotherapy.

Interestingly, E-cadherin expression was decreased (*P* = 0.006) and vimentin expression was generally increased (*P* = 0.393) in post-treatment tumor tissue compared to in matched baseline tissues (Supplementary Figure 2). However, we found no association between PD-L1 changes and morphological EMT occurrence such as sarcomatoid like change. Moreover, intervals of biopsies between baseline and after treatment was not associated with PD-L1 changes.

### Increased PD-L1 expression in response to cisplatin treatment *in vitro*

**Figure 2:**
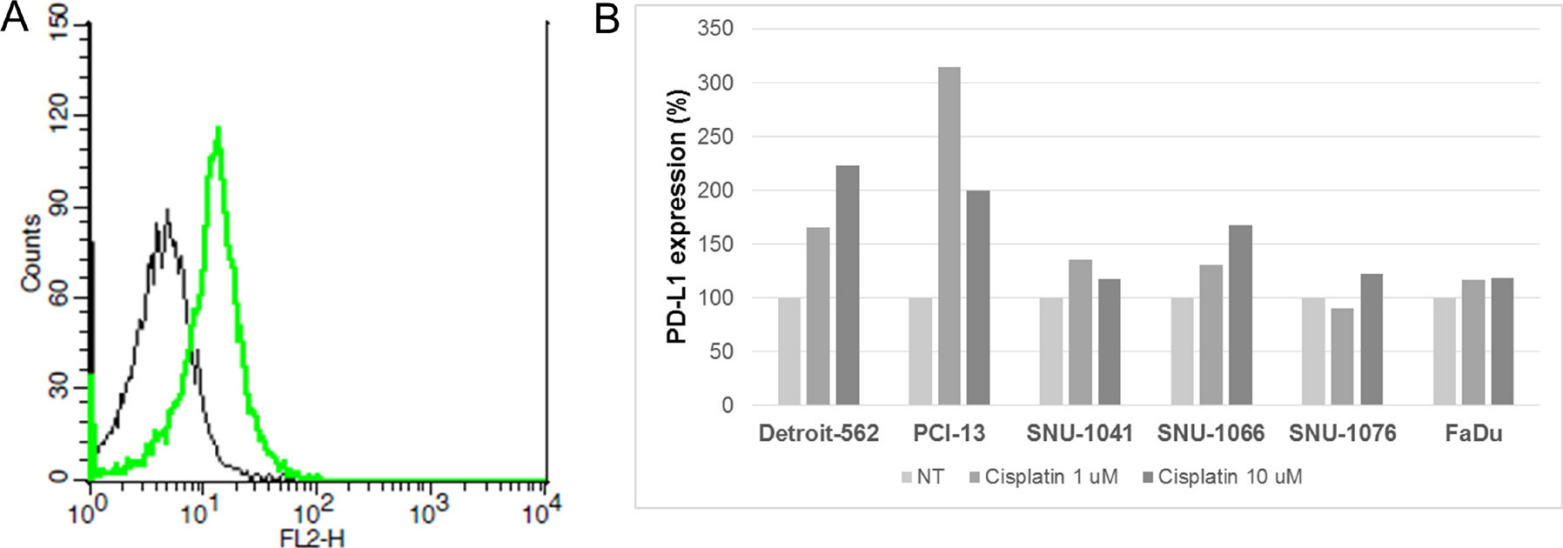
Increased PD-L1 expression by cisplatin treatment in HNSCC cells (**A**) Cisplatin 1 μM (green line) or normal saline (black line) was used to treat SNU-1041 cells for 24 h, and then flow cytometry analysis was performed using anti-PD-L1 antibody. PD-L1 expression was measured as the geometrical mean of fluorescence in gated cells. (**B**) Bar graph showing mean PD-L1 expressions according to cisplatin treatment in HNSCC cells. Each bar represents the percent change of PD-L1 expression compared to no treatment (NT) in each HNSCC cell line.

To confirm that PD-L1 expression changes following cisplatin treatment *in vitro*, HNSCC cell lines were treated with cisplatin for 24 h and PD-L1 expression after cisplatin treatment was compared with baseline expression. In all HNSCC cell lines analyzed by flow cytometry, PD-L1 expression was up-regulated after cisplatin treatment (Figure 2). This result was also observed in HNSCC cell lines treated with interferon-gamma or lipopolysaccharide, which induce PD-L1 (Supplementary Figure 3).

### Increased PD-L1 expression accompanied activation of MEK pathway

**Figure 3:**
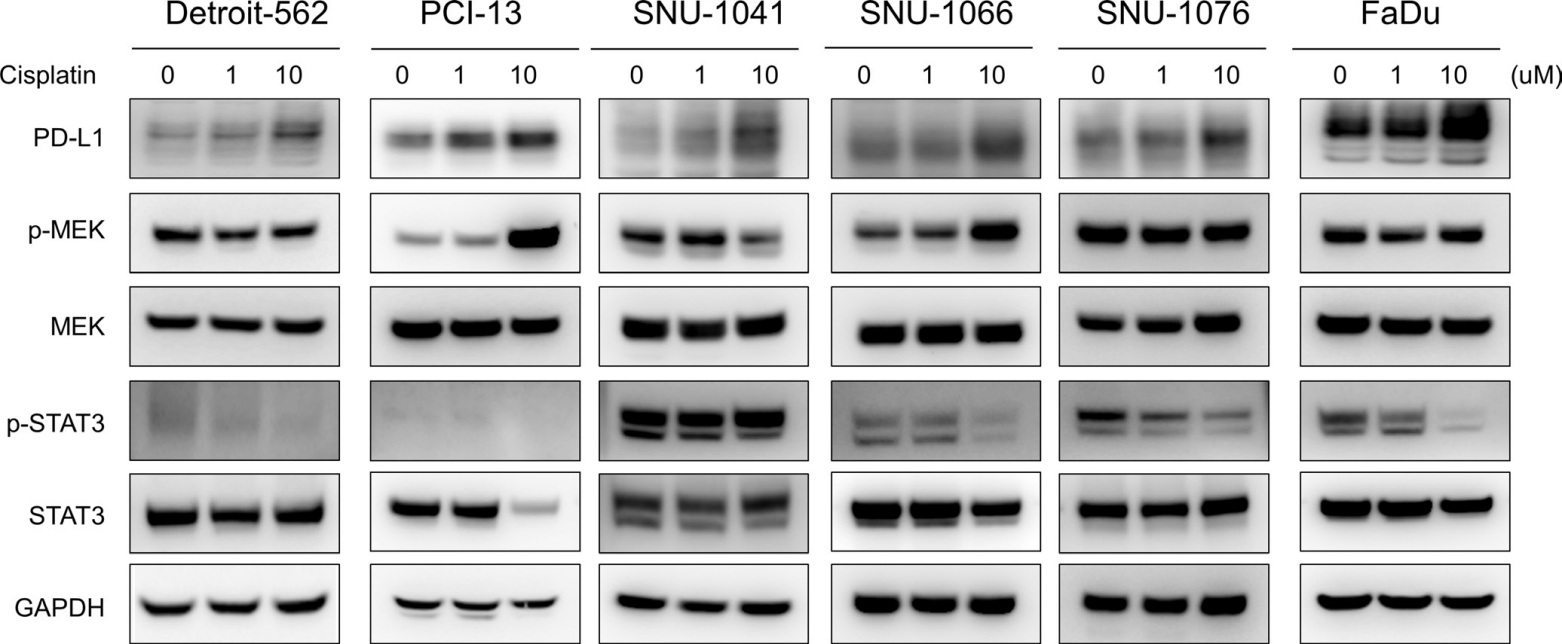
Increased PD-L1 expression by cisplatin accompanied MEK pathway activation in HNSCC cells Expression of PD-L1, phosphor-MEK (p-MEK), total MEK, phosphor-STAT3 (p-STAT3), and total STAT3 in HNSCC cells was measured by western blotting. Cisplatin treatment increased PD-L1 and p-MEK expression.

To determine which signaling pathways are related to PD-L1 up-regulation by cisplatin, western blot analysis of phospho-MEK and phospho-STAT3 was performed and PD-L1 expression was determined. PD-L1 expression increased in all HNSCC cells according to western blot analysis. Interestingly, cisplatin treatment increased the ratio of phospho-MEK/total-MEK in a dose-dependent manner in PCI-13 and SNU-1066 cells. However, the ratio of phosphor-STAT3/total-STAT3 did not increase or decrease in all HNSCC cells according to cisplatin treatment (Figure 3). We previously reported that EMT is associated with PD-L1 upregulation in gefitinib-resistance in NSCLC [13] and that EMT is associated with PD-L1 expression in HNSCC [15]; thus, we evaluated the expression of EMT markers such as E-cadherin and vimentin in cisplatin-treated HNSCC cells. However, EMT expression did not change after cisplatin treatment in HNSCC cells (Supplementary Figure 4).

## DISCUSSION

In the current study, we found that PD-L1 expression was altered after cisplatin chemotherapy in HNSCC both *in vitro* cell lines and patient samples. In HNSCC cell lines, PD-L1 up-regulation by cisplatin was accompanied by activation of the MEK pathway. In HNSCC patients, 69.2% of baseline PD-L1-negative patients became PD-L1-positive. Our results suggest that cisplatin treatment is related to PD-L1-upregulation.

The immunologic status of the host and tumor are altered during carcinogenesis and the treatment phase based on a concept of cancer immunoediting known as elimination-equilibrium-escape [17]. As PD-L1/PD-1 is an important axis conferring the immune-escape ability of tumors, PD-L1 status is continuously changed. Although *in vitro* evidence has shown that PD-L1 expression changes in the presence of various agents such as interferon-gamma, cisplatin, paclitaxel, etoposide, and 5-fluorouracil [10–12] and *in vivo* evidence has demonstrated changes in immunologic features following treatment with anti-PD-L1 inhibitors [18], few studies have confirmed whether PD-L1 changes according to chemotherapeutic agents in patient samples. Because we previously reported that PD-L1 is up-regulated in gefitinib-resistant NSCLC patients, we also compared PD-L1 status in baseline and after treatment in HNSCC patient samples. This is the first studying examining changes of PD-L1 expression in paired HNSCC patient samples.

Examining the underlying mechanism of PD-L1 up-regulation is challenging. Various reported have shown that the MEK pathway is activated according to anti-epidermal growth factor receptor treatment resistance in NSCLC, and inhibition of the MEK pathway attenuated PD-L1 up-regulation [13, 19]. Therefore, a combination of an MEK inhibitor and PD-L1 inhibitor may be synergistic [20], which has been evaluated in clinical trials. In the current study, we showed that MEK pathway activation was accompanied by PD-L1 up-regulation in cisplatin-treated HNSCC cells, indicating that MEK regulation is a crucial step in modulating PD-L1 expression in cancer.

Clinically, it is difficult to perform repeated biopsy considering bleeding risks. Therefore, assessing the immunologic profile of cancer, particularly PD-L1 status, is typically used to analyze tissues that are usually harvested by surgery or biopsy at initial diagnosis. However, as host-tumor immunologic status continuously changes, PD-L1 expression is also altered during treatment. We found that PD-L1 expression was altered in 37.1% of tumor samples after treatment. This trend was particularly strong in baseline PD-L1-negative patients (69.2%) and cisplatin-treated patients (83.3%). Previous clinical trials consistently showed that PD-L1 expression is an important biomarker for predicting anti-PD-1/PD-L1 treatment in various cancer types including HNSCC [21, 22]; thus, PD-L1 assessment should be performed in recent biopsy samples in order to precisely determine whether anti-PD-1/PD-L1 inhibitors should be used. Well-designed proof-of-concept clinical trials to confirm whether recent biopsy samples should be used for accurate biomarker analysis are needed.

There were some limitations to the current study. First, only a small number of patients was analyzed. Moreover, although nearly a half of the patients received cisplatin chemotherapy, the sequence of treatment modalities and definitive treatment were very heterogeneous. Moreover, radiotherapy would also play a role in PD-L1 up-regulation in CCRT-treated patients. However, the main finding of the study was that PD-L1 changes according to treatment, which is important because it is difficult to obtain paired biopsy samples because of technical and ethical issues. In-depth *in vitro* analysis to clearly define the association between the MEK pathway and PD-L1 up-regulation is needed.

In conclusion, PD-L1 expression in HNSCC is altered during the treatment phase, particularly following cisplatin-containing chemotherapy in baseline PD-L1-negative patients. A recent re-biopsy sample is required for precise evaluation of the PD-L1 expression status in HNSCC and to determine whether anti-PD-1/PD-L1 inhibitors should be used.

## MATERIALS AND METHODS

### HNSCC patients

Medical records were retrospectively reviewed to identify patients diagnosed with HNSCC who were treated at Seoul National University Hospital from December 2004 to November 2012. Patients from whom paraffin-embedded tumor samples were obtained prior to and after cisplatin chemotherapy were included.

### Cisplatin treatment in HNSCC patients

The decision to treat patients was determined by a multidisciplinary team [23, 24]. Patients were treated initially with induction chemotherapy and/or definitive CCRT or radical surgery (including primary tumor and regional lymph node dissection). Induction chemotherapy regimens included docetaxel, cisplatin, or 5-fluorouracil. CCRT regimens consisted of cisplatin. Radiotherapy delivered 5 days per week using a simultaneous integrated boost technique. Gross tumor lesions or high-risk volumes received 63 Gy to 67.5 Gy in 28 to 30 fractions over 6 weeks using a daily dose of 2.25 Gy, and low- and intermediate-risk volumes were irradiated to 48 Gy to 56 Gy using a daily dose of 1.8 Gy to 2.0 Gy with concurrent chemotherapy of weekly cisplatin.

### Immunohistochemistry of HNSCC patients

Representative formalin-fixed paraffin-embedded tissue blocks from each case were subjected to immunohistochemistry (IHC) using the following antibodies: mouse anti-p16 (E6H4) monoclonal antibody (mAb) (Roche/MTM/Ventana Laboratories, Tucson, AZ, USA), mouse anti–E-cadherin (36B5) mAb (Novocastra, Newcastle, UK), mouse anti-vimentin (V9) mAb (Dako, Ely, UK), and rabbit anti-PD-L1 (E1L3N) XP^®^ mAb (Cell Signaling Technology, Danvers, MA, USA). IHC was performed using the Ventana Benchmark XT system (Ventana Medical Systems). p16 was considered as positive when IHC analysis revealed diffuse and strong nuclear and cytoplasmic staining in ≥70% of tumor cells [25]. PD-L1 IHC was evaluated based on the intensity and proportion of membranes with or without cytoplasmic staining in tumor cells and was scored as follows: 0, less than 5% of tumor cells; 1, weak in ≥ 5% of tumor cells; 2, moderate in ≥ 5% of tumor cells; 3, strong in ≥ 5% of tumor cells [15, 26]. Cases showing staining for PD-L1 in ≥ 5% of tumor cells, i.e., including IHC score 1, 2 or 3, were considered as PD-L1-positive.

### Head and neck cancer cell lines experiments

Head and neck cancer cell lines were purchased from the American Type Culture Collection (Manassas, VA, USA) and Korean Cell Line Bank (Seoul, Korea) and cultured as previously described [27]. Briefly, the SNU-1066, SNU-1041 and SNU-1076 cell lines were maintained in RPMI 1640 medium containing 100 U/mL penicillin, 100 μg/mL streptomycin (Invitrogen, Carlsbad, CA, USA) supplemented with 10% fetal bovine serum (GIBCO, Grand Island, NY, USA). The Detroit-562, and FaDu and PCI-13 cell lines were maintained in American Type Culture Collection Eagle's modified essential medium (EMEM) with 100 U/mL penicillin, 100 μg/mL streptomycin (Invitrogen, Carlsbad, CA, USA) supplemented with 10% fetal bovine serum (GIBCO). All cell lines were incubated under standard culture conditions (5% CO_2_ at 37°C). Cells were resuspended in lysis buffer (Cell Signaling Technology), incubated on ice for 10 min, and centrifuged for 15 min at 4°C. Samples containing equal quantities of total protein were resolved on SDS–polyacrylamide denaturing gels, transferred to polyvinylidene fluoride membranes, and probed with antibodies according to the manufacturer's protocols. Antibodies against PD-L1, p-MEK Ser217/221, MEK, p-STAT3, STAT3, E-cadherin, vimentin, and β-actin were purchased from Cell Signaling Technology. β-Actin was used as the protein loading control Detection was performed using an enhanced Lumi-Light Western Blotting Substrate kit (Roche, Basel, Switzerland).

### Flow cytometry analysis for cell lines

Flow cytometry was performed as previously described [13]. A total of 2 × 10^5^ cells was aliquoted and placed into assay tubes. Next, 2 mL of fluorescence-activated cell sorting (FACS) buffer was added to each tube and rinsed twice by centrifugation. The cells were resuspended in 100 mL of FACS buffer with fixable viability dye (eBioscience, San Diego, CA). The cells were stained with PD-L1 phycoerythrin (PE; eBioscience) or isotype control for 30 min on ice in staining buffer (2% bovine serum albumin and 0.01% sodium azide). Analysis was conducted using a FACSCalibur instrument (BD Biosciences, Franklin Lakes, NJ, USA) with CELLQuest software (BD Biosciences). Flow cytometric analysis of PD-L1 expression in head and neck cancer cells was conducted. After 24 h, the cells were harvested and stained with either mouse anti-human PD-L1 (clone 5H1) or a mouse IgG1 isotype control followed by PE-conjugated goat anti-mouse Ig.

### Statistical analysis

The chi-square test was used to determine the nature of the associations between PD-L1 positivity and clinicopathologic parameters. Overall survival was measured from the diagnosis date until death or the last follow-up date, if censored. Survival analyses were carried out according to the Kaplan-Meier method with log-rank testing to assess differences between groups. All reported *P* values were two-sided and considered significant if *P* < 0.05. All statistical analyses were carried out using R version 3.1.2 (http://www.r-project.org).

### Ethics

The study protocol was approved by the Institutional Review Board of Seoul National University Hospital (approval number: H-1307-051-504) and was conducted in accordance with the Principles of the Declaration of Helsinki.

## SUPPLEMENTARY MATERIALS FIGURES



## References

[1] Robert C, Schachter J, Long GV, Arance A, Grob JJ, Mortier L, Daud A, Carlino MS, McNeil C, Lotem M, Larkin J, Lorigan P, Neyns B, KEYNOTE-006 investigators (2015). Pembrolizumab versus Ipilimumab in Advanced Melanoma. N Engl J Med.

[2] Topalian SL, Hodi FS, Brahmer JR, Gettinger SN, Smith DC, McDermott DF, Powderly JD, Carvajal RD, Sosman JA, Atkins MB, Leming PD, Spigel DR, Antonia SJ (2012). Safety, activity, and immune correlates of anti-PD-1 antibody in cancer. N Engl J Med.

[3] Rizvi NA, Hellmann MD, Snyder A, Kvistborg P, Makarov V, Havel JJ, Lee W, Yuan J, Wong P, Ho TS, Miller ML, Rekhtman N, Moreira AL (2015). Cancer immunology. Mutational landscape determines sensitivity to PD-1 blockade in non-small cell lung cancer. Science.

[4] Le DT, Uram JN, Wang H, Bartlett BR, Kemberling H, Eyring AD, Skora AD, Luber BS, Azad NS, Laheru D, Biedrzycki B, Donehower RC, Zaheer A (2015). PD-1 Blockade in Tumors with Mismatch-Repair Deficiency. N Engl J Med.

[5] Ock CY, Keam B, Kim S, Lee JS, Kim M, Kim TM, Jeon YK, Kim DW, Chung DH, Heo DS (2016). Pan-Cancer Immunogenomic Perspective on the Tumor Microenvironment Based on PD-L1 and CD8 T-Cell Infiltration. Clin Cancer Res.

[6] Rooney MS, Shukla SA, Wu CJ, Getz G, Hacohen N (2015). Molecular and genetic properties of tumors associated with local immune cytolytic activity. Cell.

[7] Seiwert TY, Burtness B, Mehra R, Weiss J, Berger R, Eder JP, Heath K, McClanahan T, Lunceford J, Gause C, Cheng JD, Chow LQ (2016). Safety and clinical activity of pembrolizumab for treatment of recurrent or metastatic squamous cell carcinoma of the head and neck (KEYNOTE-012): an open-label, multicentre, phase 1b trial. Lancet Oncol.

[8] Garon EB, Rizvi NA, Hui R, Leighl N, Balmanoukian AS, Eder JP, Patnaik A, Aggarwal C, Gubens M, Horn L, Carcereny E, Ahn MJ, Felip E, KEYNOTE-001 Investigators (2015). Pembrolizumab for the treatment of non-small-cell lung cancer. N Engl J Med.

[9] Herbst RS, Soria JC, Kowanetz M, Fine GD, Hamid O, Gordon MS, Sosman JA, McDermott DF, Powderly JD, Gettinger SN, Kohrt HE, Horn L, Lawrence DP (2014). Predictive correlates of response to the anti-PD-L1 antibody MPDL3280A in cancer patients. Nature.

[10] Dovedi SJ, Adlard AL, Lipowska-Bhalla G, McKenna C, Jones S, Cheadle EJ, Stratford IJ, Poon E, Morrow M, Stewart R, Jones H, Wilkinson RW, Honeychurch J, Illidge TM (2014). Acquired resistance to fractionated radiotherapy can be overcome by concurrent PD-L1 blockade. Cancer Res.

[11] Zhang P, Su DM, Liang M, Fu J (2008). Chemopreventive agents induce programmed death-1-ligand 1 (PD-L1) surface expression in breast cancer cells and promote PD-L1-mediated T cell apoptosis. Mol Immunol.

[12] Dong H, Strome SE, Salomao DR, Tamura H, Hirano F, Flies DB, Roche PC, Lu J, Zhu G, Tamada K, Lennon VA, Celis E, Chen L (2002). Tumor-associated B7-H1 promotes T-cell apoptosis: a potential mechanism of immune evasion. Nat Med.

[13] Han JJ, Kim DW, Koh J, Keam B, Kim TM, Jeon YK, Lee SH, Chung DH, Heo DS (2016). Change in PD-L1 Expression After Acquiring Resistance to Gefitinib in EGFR-Mutant Non-Small-Cell Lung Cancer. Clin Lung Cancer.

[14] Chen N, Fang W, Zhan J, Hong S, Tang Y, Kang S, Zhang Y, He X, Zhou T, Qin T, Huang Y, Yi X, Zhang L (2015). Upregulation of PD-L1 by EGFR Activation Mediates the Immune Escape in EGFR-Driven NSCLC: Implication for Optional Immune Targeted Therapy for NSCLC Patients with EGFR Mutation. J Thorac Oncol.

[15] Ock CY, Kim S, Keam B, Kim M, Kim TM, Kim JH, Jeon YK, Lee JS, Kwon SK, Hah JH, Kwon TK, Kim DW, Wu HG (2016). PD-L1 expression is associated with epithelial-mesenchymal transition in head and neck squamous cell carcinoma. Oncotarget.

[16] Wu CT, Chen WC, Chang YH, Lin WY, Chen MF (2016). The role of PD-L1 in the radiation response and clinical outcome for bladder cancer. Sci Rep.

[17] Schreiber RD, Old LJ, Smyth MJ (2011). Cancer immunoediting: integrating immunity's roles in cancer suppression and promotion. Science.

[18] Tumeh PC, Harview CL, Yearley JH, Shintaku IP, Taylor EJ, Robert L, Chmielowski B, Spasic M, Henry G, Ciobanu V, West AN, Carmona M, Kivork C (2014). PD-1 blockade induces responses by inhibiting adaptive immune resistance. Nature.

[19] Ota K, Azuma K, Kawahara A, Hattori S, Iwama E, Tanizaki J, Harada T, Matsumoto K, Takayama K, Takamori S, Kage M, Hoshino T, Nakanishi Y, Okamoto I (2015). Induction of PD-L1 Expression by the EML4-ALK Oncoprotein and Downstream Signaling Pathways in Non-Small Cell Lung Cancer. Clin Cancer Res.

[20] Ebert PJ, Cheung J, Yang Y, McNamara E, Hong R, Moskalenko M, Gould SE, Maecker H, Irving BA, Kim JM, Belvin M, Mellman I (2016). MAP Kinase Inhibition Promotes T Cell and Anti-tumor Activity in Combination with PD-L1 Checkpoint Blockade. Immunity.

[21] Ferris RL, Blumenschein G, Fayette J, Guigay J, Colevas AD, Licitra L, Harrington K, Kasper S, Vokes EE, Even C, Worden F, Saba NF, Iglesias Docampo LC (2016). Nivolumab for Recurrent Squamous-Cell Carcinoma of the Head and Neck. N Engl J Med.

[22] Chow LQ, Haddad R, Gupta S, Mahipal A, Mehra R, Tahara M, Berger R, Eder JP, Burtness B, Lee SH, Keam B, Kang H, Muro K Antitumor Activity of Pembrolizumab in Biomarker-Unselected Patients With Recurrent and/or Metastatic Head and Neck Squamous Cell Carcinoma: Results From the Phase Ib KEYNOTE-012 Expansion Cohort. J Clin Oncol.

[23] Lim Y, Keam B, Koh Y, Kim TM, Lee SH, Hah JH, Kwon TK, Kim DW, Wu HG, Sung MW, Heo DS, Kim KH (2013). Clinical outcomes of radiation-based locoregional therapy in locally advanced head and neck squamous cell carcinoma patients not responding to induction chemotherapy. Oral Surg Oral Med Oral Pathol Oral Radiol.

[24] Ock CY, Keam B, Lim Y, Kim TM, Lee SH, Kwon SK, Hah JH, Kwon TK, Kim DW, Wu HG, Sung MW, Heo DS (2016). Effect of induction chemotherapy on survival in locally advanced head and neck squamous cell carcinoma treated with concurrent chemoradiotherapy: single center experience. Head Neck.

[25] Grønhøj Larsen C, Gyldenløve M, Jensen DH, Therkildsen MH, Kiss K, Norrild B, Konge L, von Buchwald C (2014). Correlation between human papillomavirus and p16 overexpression in oropharyngeal tumours: a systematic review. Br J Cancer.

[26] Kim MY, Koh J, Kim S, Go H, Jeon YK, Chung DH (2015). Clinicopathological analysis of PD-L1 and PD-L2 expression in pulmonary squamous cell carcinoma: comparison with tumor-infiltrating T cells and the status of oncogenic drivers. Lung Cancer.

[27] Keam B, Kim S, Ahn YO, Kim TM, Lee SH, Kim DW, Heo DS (2015). *In vitro* anticancer activity of PI3K alpha selective inhibitor BYL719 in head and neck cancer. Anticancer Res.

