# Supplementary material for: Changes in programmed death-ligand 1 expression during cisplatin treatment in patients with head and neck squamous cell carcinoma
Source: Oncotarget. 2017 Jun 16;8(58):97920–7. doi: 10.18632/oncotarget.18542 (PMC5716702; doi:10.18632/oncotarget.18542)
Supplement: Supplementary file 1 [file oncotarget-08-97920-s001.pdf]

Changes in programmed death-ligand 1 expression during cisplatin treatment in patients with head and neck squamous cell carcinoma

Supplementary Materials

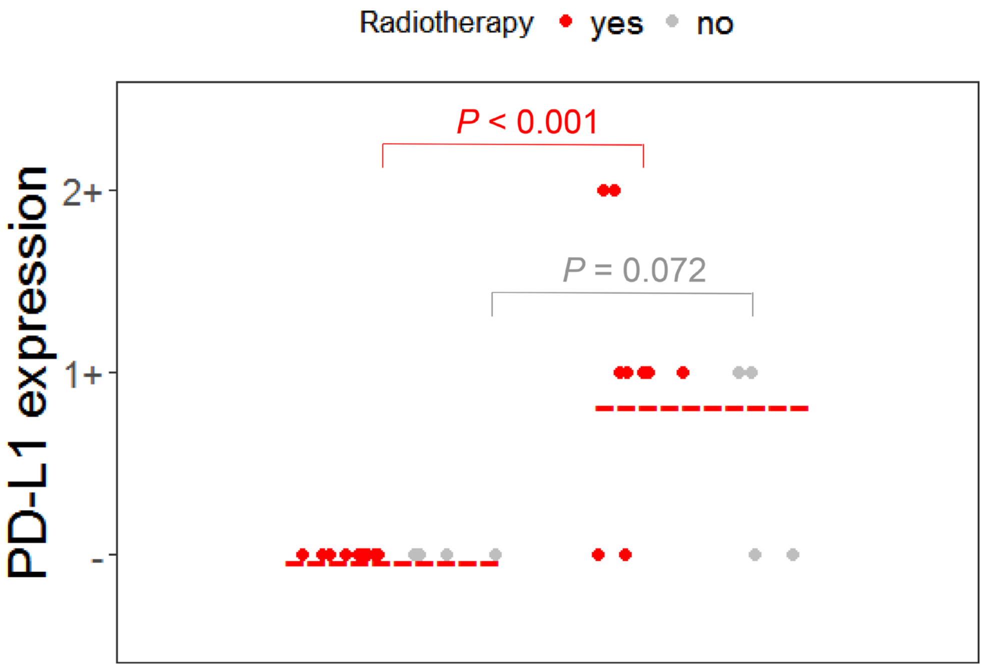

Supplementary Figure 1: The change of PD-L1 according to radiotherapy in PD-L1-negative HNSCC patients. Among baseline PD-L1-negative patients, PD-L1 expression was more significantly up-regulated in patients with radiotherapy (red dot) compared to those who were not exposed to radiotherapy (grey dot).

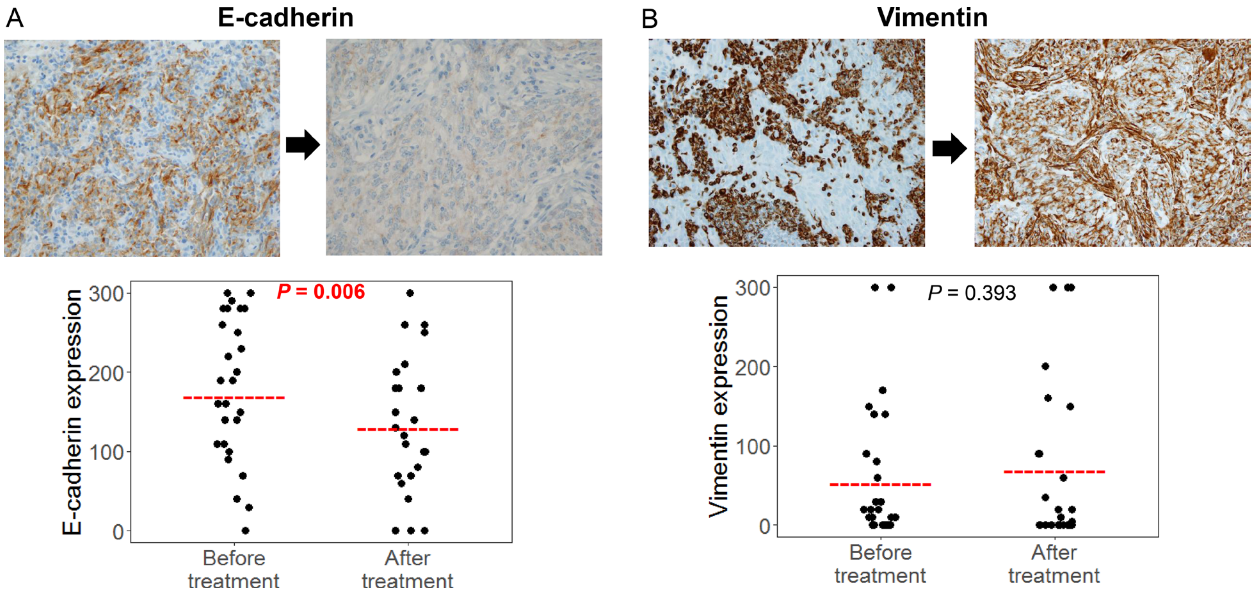

Supplementary Figure 2: Changes of EMT markers before and after treatment. Representative cases of e-cadherin down-regulation (**A**, ×400, top) and vimentin up-regulation (**B**, ×400, top) after treatment in HNSCC patient samples. Each dot represented E-cadherin (A) or vimentin (B) expression positivity, and red bar represented mean e-cadherin or vimentin expression positivity of each group.

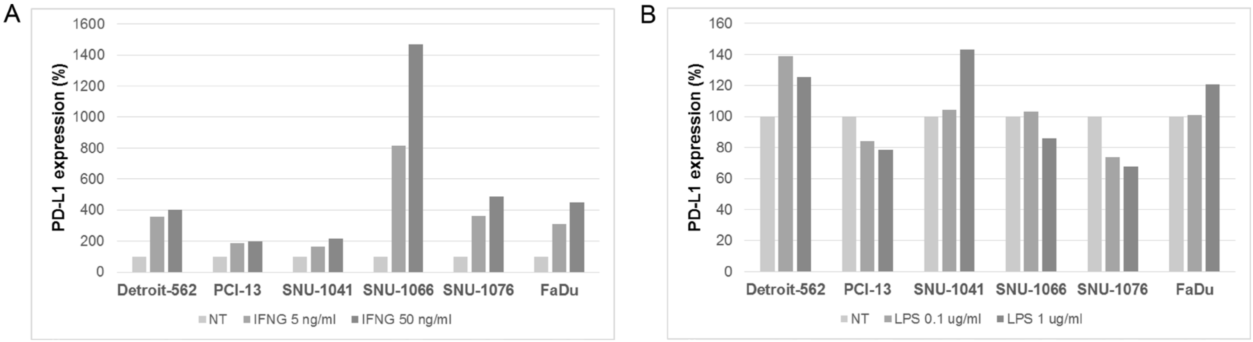

Supplementary Figure 3: PD-L1 expression is increased by interferon-gamma (IFNG) or lipopolysaccharide (LPS) inhead and neck squamous cancer cells. PD-L1 expressions measured by flow cytometry were compared according to no treatment (light grey), IFNG 5 ng/ml (**A**) or LPS 1 ug/ml (**B**, grey), and IFNG 50 ng/ml (A) or LPS 1 ug/ml (B, dark grey) in each head and neck squamous cancer cells.

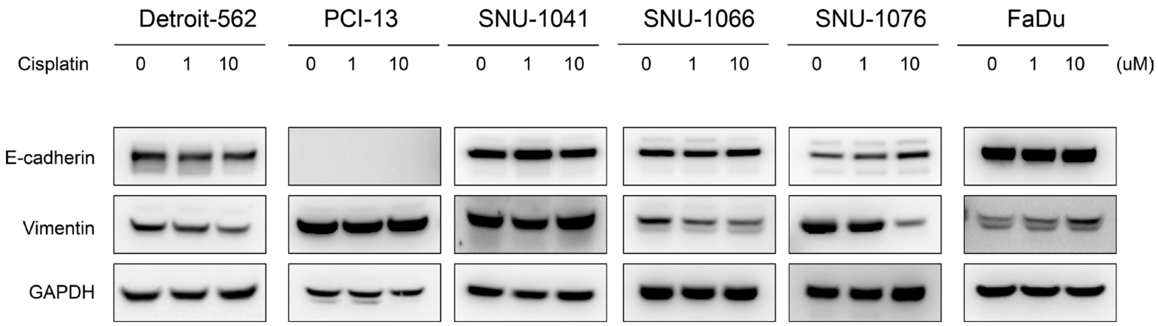

Supplementary Figure 4: Association of PD-L1 expression and epithelial-mesenchymal transitions (EMT) markers in head and neck squamous cancer cells. E-cadherin and vimentin expressions measured by western blot were compared according to cisplatin treatment in head and neck squamous cancer cells.
